# Steel Fiber-Reinforced Concrete: A Systematic Review of the Research Progress and Knowledge Mapping

**DOI:** 10.3390/ma15176155

**Published:** 2022-09-05

**Authors:** Muhammad Nasir Amin, Waqas Ahmad, Kaffayatullah Khan, Ayaz Ahmad

**Affiliations:** 1 Department of Civil and Environmental Engineering, College of Engineering, King Faisal University, Al-Ahsa 31982, Saudi Arabia; 2 Department of Civil Engineering, COMSATS University Islamabad, Abbottabad 22060, Pakistan; 3 MaREI Centre, Ryan Institute and School of Engineering, College of Science and Engineering, National University of Ireland Galway, H91 TK33 Galway, Ireland

**Keywords:** concrete, steel fibers, steel fiber-reinforced concrete, scientometric analysis

## Abstract

This study performed a scientometric-based examination of the literature on steel fiber-reinforced concrete (SFRC) to identify its key elements. Typical review papers are limited in their capacity to link distinct segments of the literature in an organized and systematic method. The most challenging aspects of current research are knowledge mapping, co-occurrence, and co-citation. The Scopus search engine was used to search for and obtain the data required to meet the goals of the study. During the data evaluation, the relevant publication sources, keyword assessment, productive authors based on publications and citations, top papers based on citations received, and areas vigorously involved in SFRC studies were recognized. The VOSviewer software tool was used to evaluate the literature data from 9562 relevant papers, which included citation, abstract, bibliographic, keywords, funding, and other information. Furthermore, the applications and constraints related to the usage of SFRC in the construction sector were examined, as well as potential solutions to these constraints. It was determined that only 17 publication sources (journals/conferences) had published at least 100 articles on SFRC up to June 2022. Additionally, the mostly employed keywords by authors in SFRC research include steel fibers, fiber-reinforced concrete, concrete, steel fiber-reinforced concrete, and reinforced concrete. The assessment of authors revealed that 39 authors had published at least 30 articles. Moreover, China, the United States, and India were found to be the most active and participating countries based on publications on SFRC research. This study can assist academics in building collaborative initiatives and communicating new ideas and techniques because of the quantitative and graphical depiction of participating nations and researchers.

## 1. Introduction

Concrete is a composite, brittle material utilized in a variety of engineering structures, including foundations, pavements, tunnels, bridges, walls, reservoirs, and dams [1,2,3]. Because of these numerous uses, a great deal of research has been conducted to enhance the characteristics of concrete for greater applicability [4,5,6,7,8,9,10]. The addition of steel to concrete was one of many attempts to improve its performance [11,12,13,14]. These traditional reinforcements are steel reinforcing bars positioned at precise locations inside the structure to resist applied tensile and shear stresses [15,16]. In contrast, the integration of steel fibers is often discontinuous and random in the concrete mixture [17,18,19]. For concrete mixtures in which the fibers are evenly scattered, they serve a significant role in reducing the incidence of cracks caused by variations in relative humidity and temperature [20,21,22,23]. The addition of fibers (steel/natural/synthetic) to the cementitious mixtures improves their mechanical properties [24,25,26,27,28]. Although the role of fiber might not always involve an increase in strength, it has a considerable favorable effect on ductility, resilience to dynamic loading, and toughness [29,30,31,32,33,34,35]. Concrete reinforcement with fibers is not a substitute for traditional steel reinforcing bars because steel bars and fibers serve distinctive, but both function in improving the concrete performance [36,37,38]. Steel fiber-reinforced concrete (SFRC) refers to the inclusion of short, discontinuous steel fibers into the matrix of concrete after mixing [39,40,41]. Water, aggregates, cement, and steel fibers are the primary components of SFRC [42,43,44]. Depending on the intended uses, SFRC may additionally comprise pozzolana and admixtures [45,46,47,48,49,50,51,52]. 

As scientists conduct more investigation on the SFRC in response to rising concerns about the brittle nature of concrete, researchers confront knowledge restraints that may inhibit new academic and research partnerships. Hence, it is critical to build and execute a system that allows academics to acquire crucial knowledge from the best reputable sources available. A scientometric technique using the software application may assist in solving this problem. Recently, several review studies have been performed on SFRC [53,54,55,56], but these are traditional reviews. This study intends to perform a scientometric examination of bibliometric data available on SFRC research up to June 2022. Employing a necessary software, a scientometric assessment may perform a quantified evaluation of massive bibliographic data. Traditional review papers are incapable of correctly and thoroughly linking various sections of the literature. Scientific visualization, co-occurrence, and co-citations are all complex components of advanced investigations [57,58,59]. The aim of scientometric analysis in this study is to identify the sources with the most publications, the authors with the most papers and citations, the top cited articles, and the regions keenly participating in a research topic. The Scopus search engine was used to acquire data from 9562 relevant publications comprising abstracts, citations, keywords, bibliographic, funding, and other information, which were then evaluated using the VOSviewer tool. In addition, the applications and limits associated with the use of SFRC in the construction sector, as well as potential solutions to these constraints, were discussed. This study will assist academia in building joint undertakings and exchanging novel thoughts and methodologies as a consequence of the graphical and statistical depiction of researchers and nations.

## 2. Review Strategy

This work used scientometric evaluation of bibliometric data [60,61,62] to quantify the many aspects of the literature. Scientific mapping, a method built by specialists for bibliographic records processing, is used in scientometric investigations [63,64]. Many papers have been recorded on the topic under study; hence, it is critical to use a reliable database. Web of Science and Scopus are two highly reliable databases that are ideal for this reason [65,66]. Scopus, which is strongly advised by scholars [67,68], was utilized to gather bibliographic data on SFRC research. A Scopus search for the keyword “steel fiber reinforced concrete” returned 18,481 items as of June 2022. To minimize extraneous papers, many filter settings were used. Table 1 indicates the complete method of data extraction, evaluation, and the different limits/filters used during the study. Furthermore, numerous investigations have documented a similar approach in different fields of study [69,70,71,72]. After applying these restrictions to the Scopus database, 14,110 items remained. Scopus records were stored in Comma Separated Values (CSV) format for further screening. It was found that the files retrieved also contained the data on fiber-reinforced polymers, which were removed manually, and only SFRC data comprising 9562 items were used for further analysis using the software tool. The scientific representation and quantitative assessment of the received information were built using VOSviewer (version 1.6.18). VOSviewer is a publicly accessible, open-source mapping product that is regularly used in numerous research disciplines and proposed by academics [73,74]. Therefore, the present study’s aims were fulfilled by using VOSviewer. The resultant data (CSV files) were imported into VOSviewer, and analysis was carried out while data consistency and reliability were maintained. The publishing sources, the most often occurring keywords, the researchers with the most published papers and citations, the documents with the most citations, and the state’s engagement were all analyzed during the scientometric study. Graphs were presented to depict the distinct attributes, their interrelationships, and co-occurrence, while tables were constructed to display quantitative data.

## 3. Results and Discussions

### 3.1. Research Progress 

**Figure 1:**
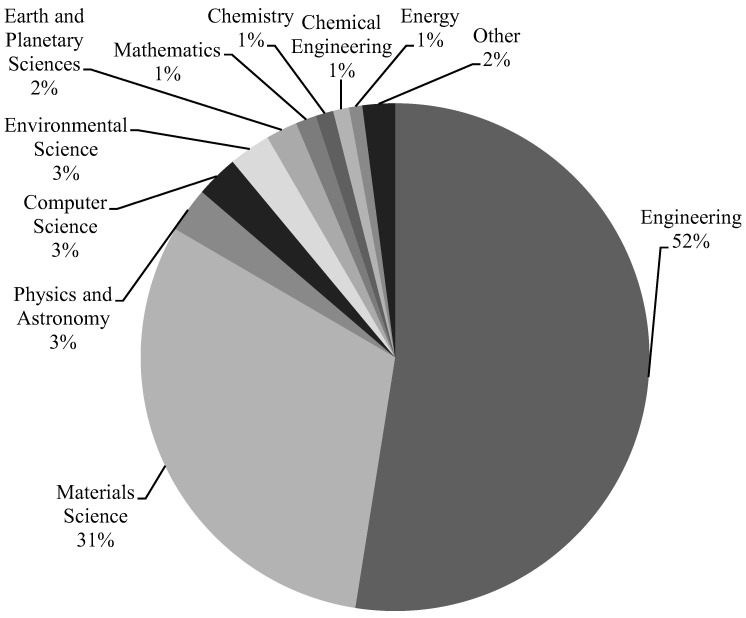
Documents’ subject area in the research of SFRC.

**Figure 2:**
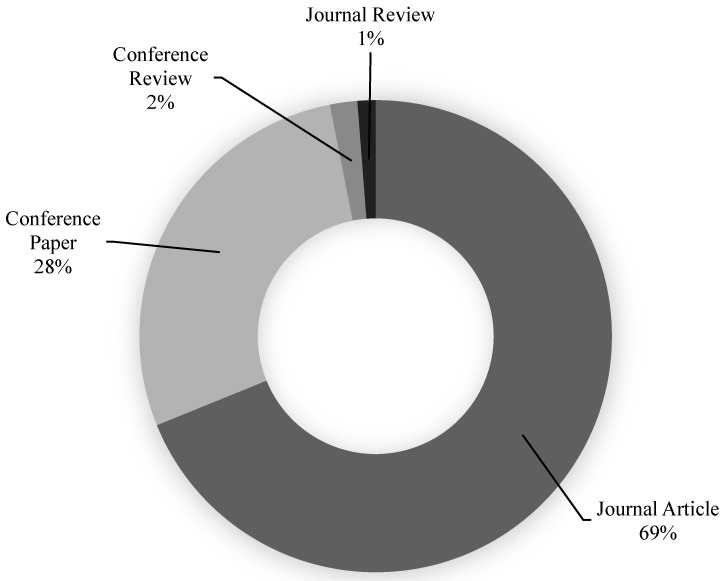
Types of documents published on the SFRC research up to June 2022.

The Scopus analyzer was used to find the most pertinent research fields for this assessment. Engineering and Materials Science were judged to be the top two document-generating fields, with about 52% and 31% of papers, respectively, providing a total of 83% of documents in the SFRC study, as shown in Figure 1. Furthermore, the Scopus database was searched for types of publications on the topic study field (Figure 2). Based on this analysis, journal papers, conference articles, conference reviews, and journal reviews comprised around 69%, 28%, 2%, and 1% of all data, respectively. As the first paper on the SFRC research was found in 1972, Figure 3 displays the yearly development of papers published in the present study field from 1972 to June 2022. Until the year 2000, there was modest growth in the number of publications in the field of SFRC studies, with an average of roughly 50 articles published each year. Following then, the number of articles steadily increased, with an average of almost 359 articles each year between 2001 and 2015, with 710 articles in 2015. Between 2016 and 2021, the number of publications increased markedly, with an average of 1101 articles annually between 2016 and 2021, with 1289 publications in 2021. The number of publications on the topic study field is rising year by year, with 668 published so far this year (June 2022).

### 3.2. Mapping Publication Sources

**Figure 4:**
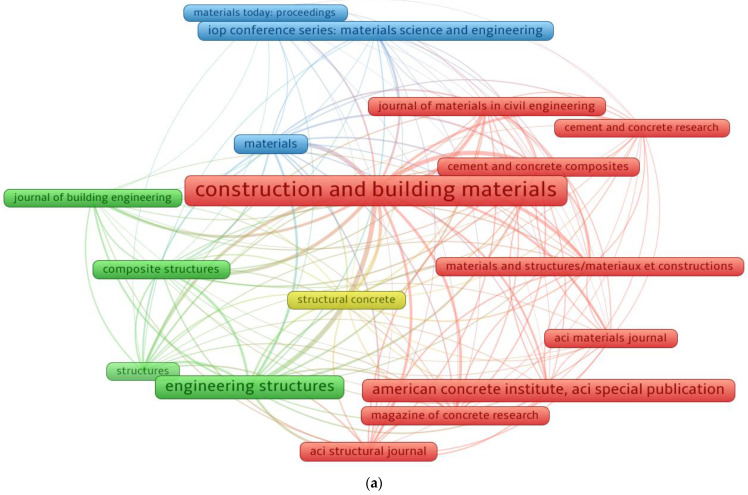

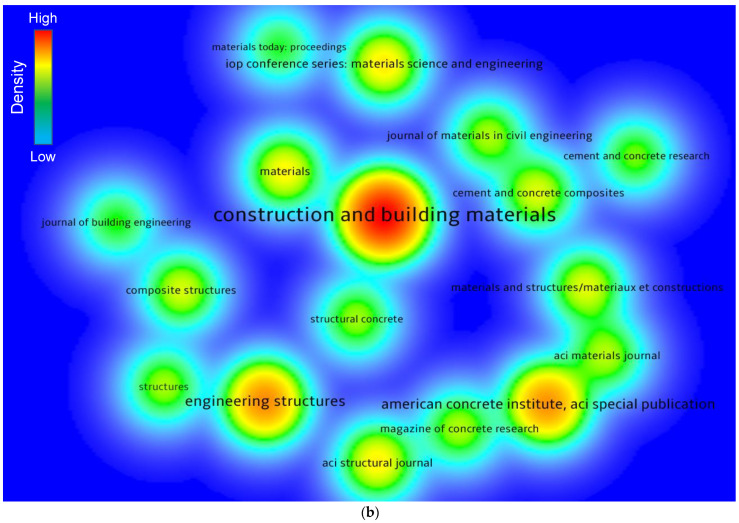
Mapping of publication sources with at least 100 publications: (**a**) Network map; (**b**) Density map.

A VOSviewer software was used to evaluate publication outlets (journals/conferences) based on bibliographic data. A minimum of 100 articles limit for a source was set, and 17 of the 1115 sources met this condition. Table 2 displays the sources that contain at least 100 papers on SFRC research up to June 2022, as well as the number of citations received during that time. The top three publishing sources, based on total publications, were discovered to be “Construction and building materials (CBM)”, “Engineering structures”, and “American concrete institute, ACI special publication”, with 877, 420, and 403 documents, respectively. Furthermore, the top three sources-based citations received up to June 2022 are “CBM”, which received 29,863, “Cement and concrete research”, which received 9749, and “Cement and concrete composites”, which received 9708 citations. This analysis, in particular, would lay the framework for future scientometric evaluations in SFRC research. Furthermore, earlier typical review studies were unable to provide a systematic overview of the literature. Figure 4 shows an image of sources that have published at least 100 articles. Based on document count, the frame dimension in Figure 4a is related to the outlet’s influence on the research field under study; a larger frame size suggests a stronger impact. As an example, “CBM” has a wider frame than the others, indicating that it is a journal of significant value in the current study field. Four groups/clusters were constructed, each with its own color on the map (blue, yellow, red, and green). The extent of the research outlet or the frequency with which they are co-cited in related papers is used to form groups/clusters [75]. The VOSviewer classified sources based on their co-citation rates in publications. The red cluster, for example, comprises nine items that have been co-cited many times in the same papers. Additionally, the connections between close frames (publishing sources) in a group/cluster are stronger than those between widely dispersed frames. As an example, “CBM” has a stronger correlation with “Cement and concrete composites” than with “ACI materials journal”. As shown in Figure 4b, different colors correlate to different density concentrations for a journal/conference. Red has the largest concentration of density, followed by yellow, green, and blue. “CBM” and “Engineering structures” are highlighted in red, indicating a greater contribution to SFRC research. This finding will assist academics in selecting reputable publication sources for the purpose of literature review and targeting for their new research publications.

### 3.3. Mapping Keywords

**Figure 5:**
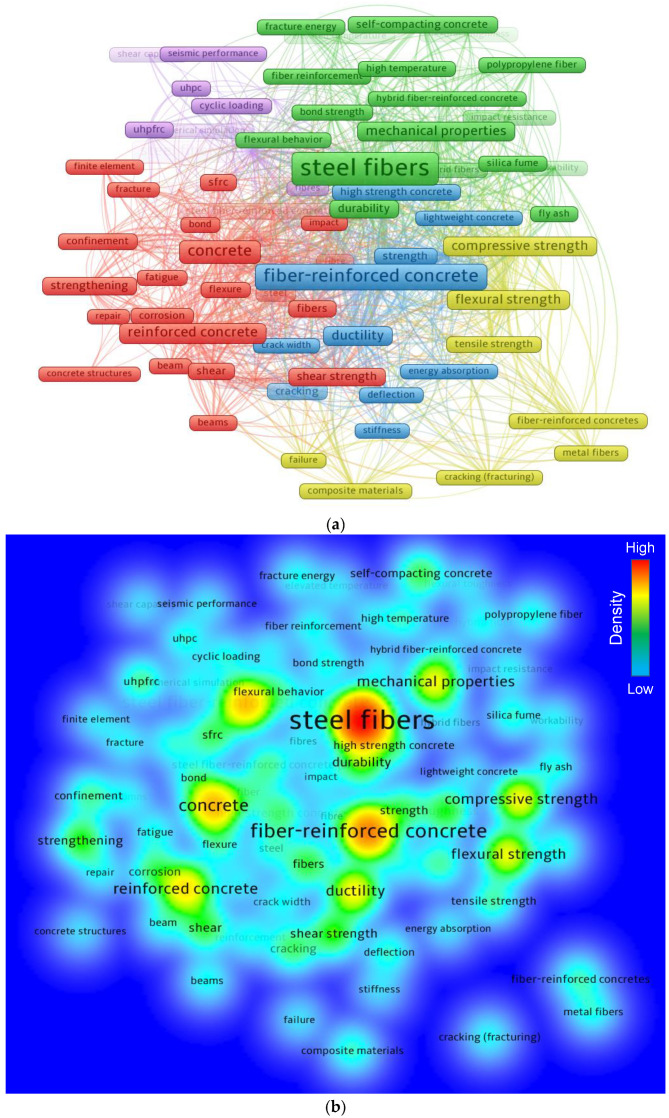
Mapping keywords in the subject topic: (**a**) Scientific map; (**b**) Density map.

Keywords are important in research since they differentiate and highlight the primary subject of the research area [76]. The lowest repeating threshold for a keyword was maintained at 50, and 81 of the 12,955 keywords were retained. Table 3 displays the leading 30 keywords that appear most regularly in the literature. Steel fibers, fiber-reinforced concrete, concrete, steel fiber-reinforced concrete, and reinforced concrete are the five highly regularly occurring keywords in the SFRC research. Based on the keyword assessment, the insertion of steel fibers in concrete has mostly been studied to enhance the mechanical and durability performance of concrete, particularly to control brittleness, resist crack propagation through crack bridging, and increase ductility. Figure 5 depicts a systematic graph of keywords with co-occurrences, linkages, and density proportionate to their rate of occurrence. The dimensions of a keyword’s frame in Figure 5a show its frequency, while its location shows its co-occurrence in articles. Furthermore, the map shows that the leading keywords have bigger frames than the others, implying that these are critical keywords for critical analysis in SFRC studies. The map emphasizes groups/clusters in a way that demonstrates their co-occurrence in a range of published articles. The co-occurrence of multiple terms in articles determines the color-coded grouping. Figure 5a depicts five clusters in various hues. Different colors reflect different keyword density concentrations, as seen in Figure 5b. The colors red, yellow, green, and blue are sorted in order of density strength, with red having the highest density concentration and blue having the lowest. Steel fibers, fiber-reinforced concrete, concrete, and other notable keywords are highlighted in red or yellow, suggesting a higher density of occurrences. This finding will support ambitious scholars in selecting keywords that will aid in recognition of available papers on a particular topic.

### 3.4. Mapping Authors

**Figure 6:**
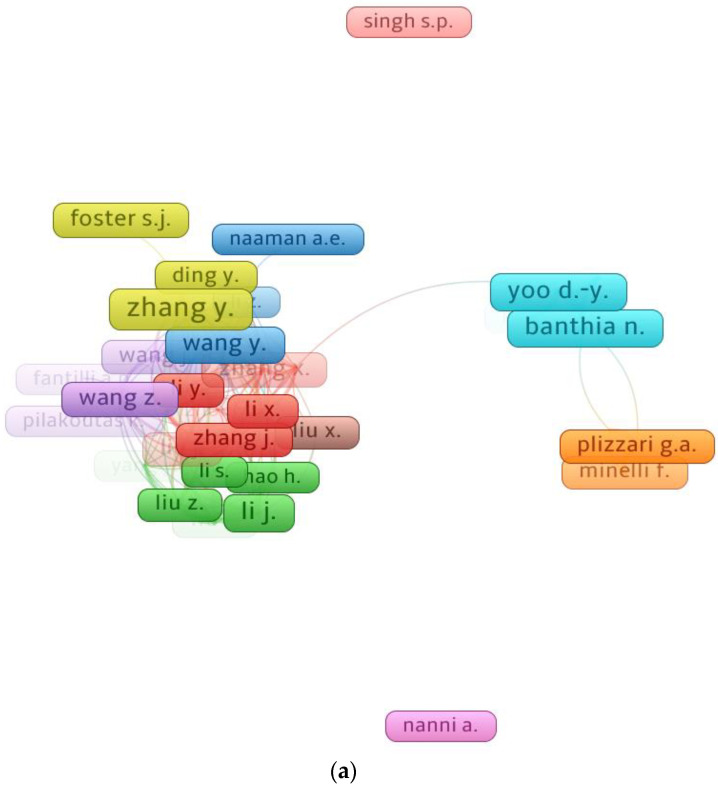

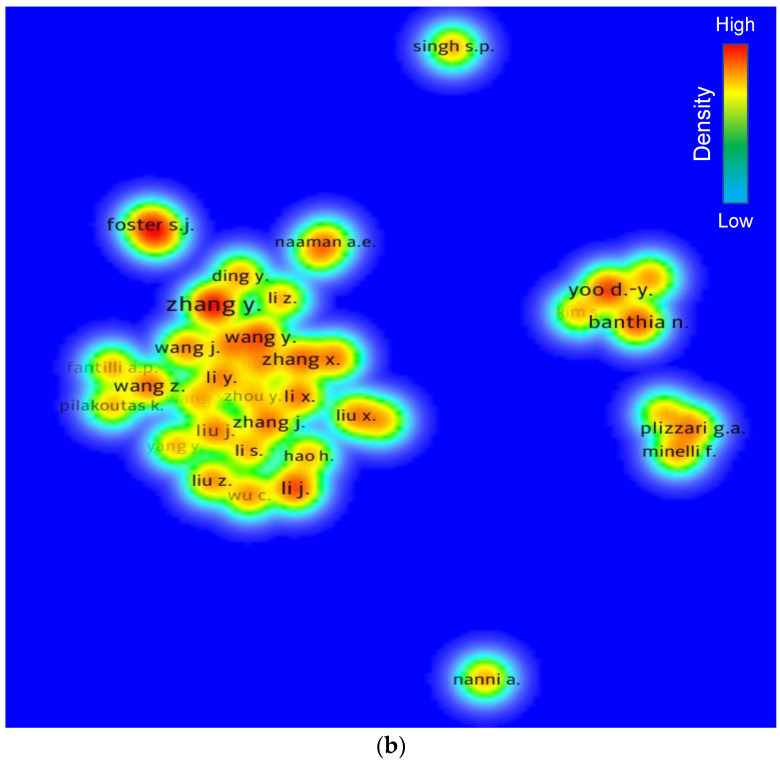
Scientific collaboration of authors: (**a**) Map of authors with minimum 30 publications; (**b**) Density of authors based on their contribution.

Citations demonstrate a scientist’s significance in a certain field of research [77]. The minimum paper criteria for a researcher was set at 30, and 39 of 14,611 researchers met this condition. Table 4 shows the authors with the most papers and citation count in the field of SFRC, as revealed using bibliographic data and VOSviewer. The average citations for a writer were computed by dividing the total number of citations by the total number of papers. It is complicated to evaluate a researcher’s performance when all indicators, like the number of documents, average citations, and total citations, are considered. Alternatively, the researcher’s rating will be assessed separately for each component. According to the data analysis, Zhang Y. is the most productive researcher, with 86 publications, followed by Yoo D.-Y. with 66 and Li J. with 63 publications. Yoo D.-Y. leads the field in total citations with 2944, Banthia N. is second with 2689, and Naaman A.E. is third, with 2269 citations in the present research domain. Additionally, when the average number of citations is compared, Naaman A.E. may be at the top with almost 71 average citations, Shah S.P. may be second with about 47, and Yoo D.-Y. may be third with around 45 average citations. Figure 6 displays the relationship between writers having at least 30 publications and the most well-known authors. The scientific mapping of researchers who have contributed at least 30 publications to the present field of study is depicted in Figure 6a. Based on citations, 37 of the 39 authors form the greatest group of connected writers. According to our analysis, the majority of SFRC researchers are related by citations. Moreover, Figure 6b displays that the leading authors in the present research area have higher density concentrations based on number of publications.

### 3.5. Mapping Documents

**Figure 7:**
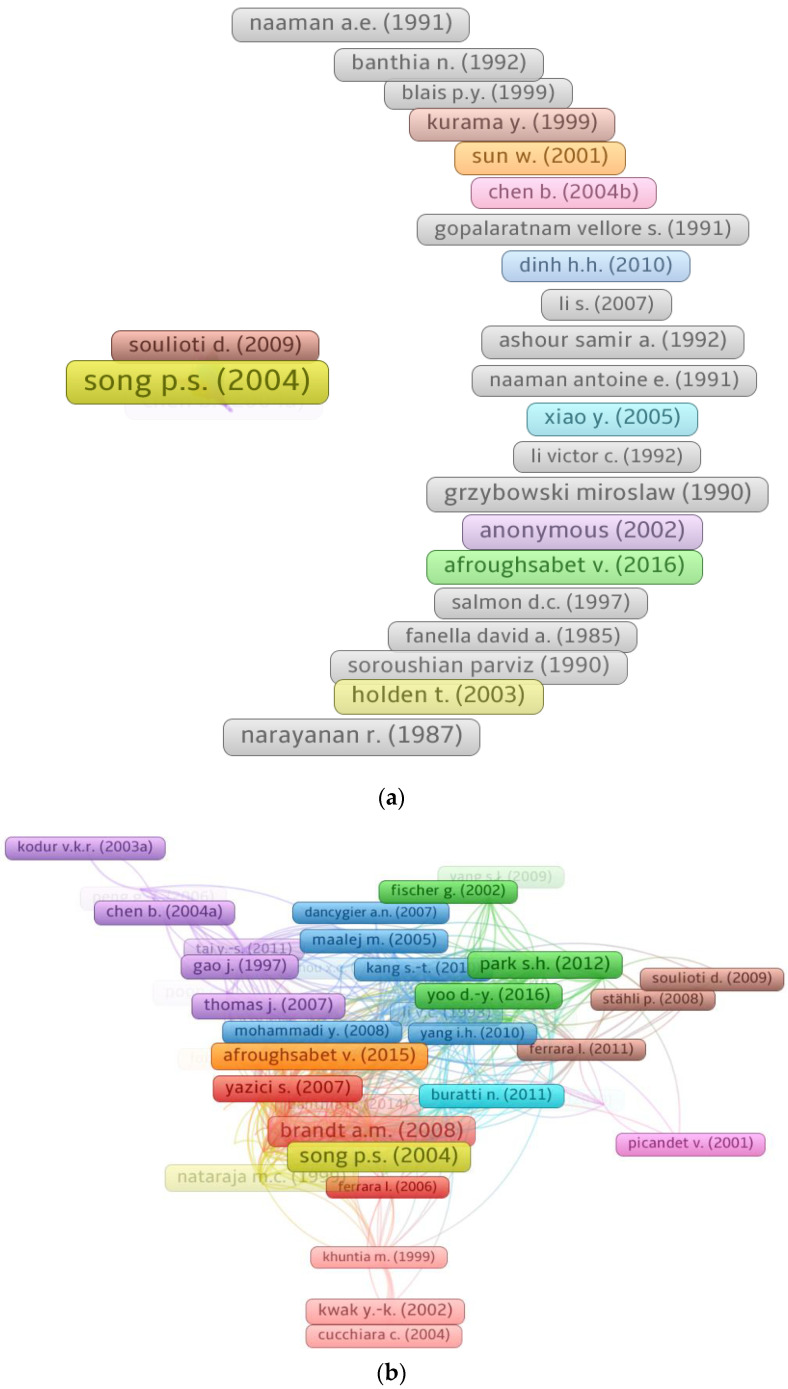
Knowledge map of documents: (**a**) Map of documents with at least 150 citations; (**b**) Connected documents based on citations.

The number of citations received by an article indicates its significance in a certain academic topic. Papers with a high number of citations are considered to be pioneering in their respective academic fields. The minimum number of citations for a document was set at 150, and 123 of 9562 publications satisfied this requirement. Table 5 includes the top five documents in the area of SFRC based on citations, as well as their authors and citation counts. Song P.S.’s [78] paper “Mechanical properties of high-strength steel fiber-reinforced concrete” has received 593 citations. Brandt A.M. [79] and Park S.H. [80] acquired 552 and 406 citations, respectively, for their works, placing them in the top three. However, only 60 publications had received more than 200 citations as of June 2022. Furthermore, Figure 7 illustrates the scientific visualization of papers and their connections to the subject of the current study based on citations. Figure 7a depicts a map of documents with at least 150 citations up to June 2022. Figure 7b depicts the scientific map of papers linked by citations. Citations connected 100 of 123 articles, according to the data assessment. As a result, citations connect the majority of the prominent papers in the current research domain.

### 3.6. Mapping Countries

**Figure 8:**
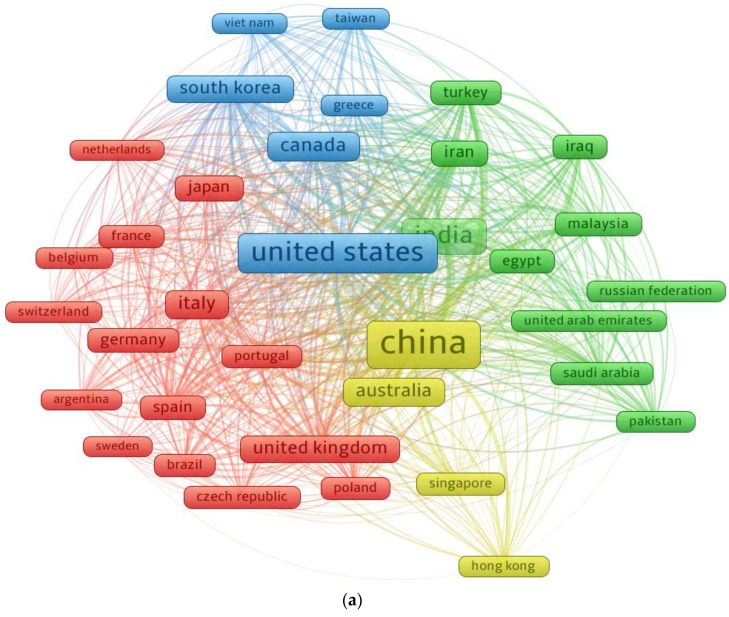

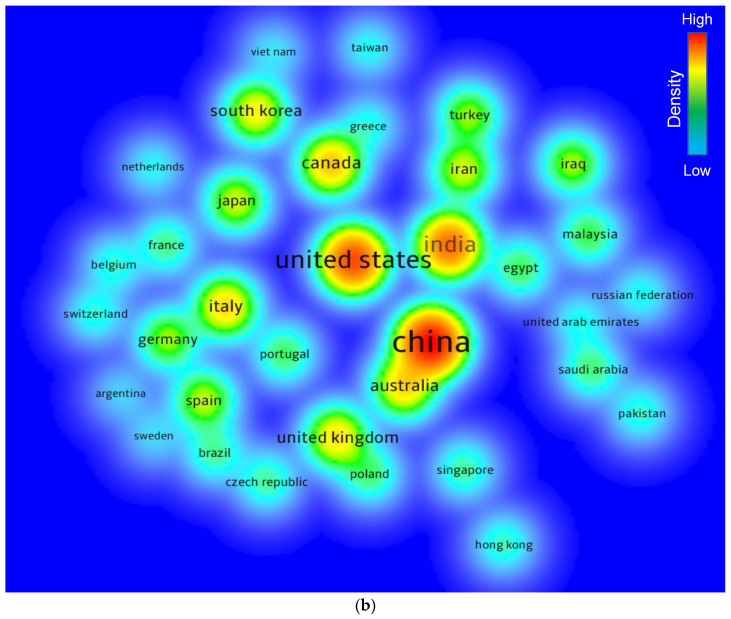
Mapping of countries with minimum 50 publications: (**a**) Scientific map; (**b**) Density.

Various states have participated with more materials in the present research topic than others, and they intend to continue doing so. The systematic graph was built so that readers may look at areas dedicated to SFRC research. The minimum number of papers that a country may possess was set at 50, and 35 countries met this requirement. Table 6 shows that the nations represented have published at least 50 documents on the current study topic. China, the United States, and India published the most papers, with 1607, 1139, and 923 papers, respectively. Furthermore, the United States obtained 24,770 citations, China received 22,924 citations, and Italy received 12,190 citations. The systematic map and the density strength of nations linked by citations are depicted in Figure 8. The size of a frame in Figure 8a is proportionate to a country’s effect on the topic studied based on the number of documents published. Figure 8b displays that the nations with the highest degree of involvement had a higher density. The graphical interpretation and quantitative record of the participating countries will help young scientists form scientific alliances, create joint companies, and exchange new approaches and thoughts. Scholars from nations interested in conducting SFRC studies can cooperate with specialists in the field and benefit from their experience.

## 4. Discussions and Applications of SFRC

This systematic study performed statistical analysis and mapping of the SFRC research using bibliographic data. Previous manual review studies lacked the ability to link distinct regions of the literature entirely and accurately. This study found the publication’s sources (journals/conferences) that published the most documents, the most often used keywords in publications, the documents and researchers with the greatest citations, and the nations that were actively engaged in SFRC research. According to keyword analysis, the incorporation of steel fibers in concrete has mostly been explored to improve the mechanical and durability performance of concrete, especially to reduce brittleness, resist fracture propagation by crack bridging, and increase ductility. Furthermore, the literature and its citations were analyzed to identify highly devoted and participating nations based on publication count. The graphical depiction and quantitative analysis of participating nations and researchers will aid young scientists in making scientific collaborations, instituting joint ventures, and exchanging new methodologies and concepts. Scholars from nations intent on extending research on the use of SFRC can cooperate with experts in the field and benefit from their knowledge.

SFRC has been utilized in a variety of applications, including bridges, tunnel linings, retaining walls, road pavements, slabs, hydraulic structures, shotcrete, foundations, and precast members [83,84,85,86,87,88,89,90,91,92]. SFRC is helpful in roadway pavement and precast members because of its high flexural strength, reduced total pavement thickness, and increased resilience to impact and repetitive loads [93,94,95,96]. When utilized in hydraulic constructions, SFRC has a strong resistance to eroding induced by the rapid passage of water [97,98,99]. Fiber shotcretes are particularly effective for rock slope stability, tunnel lining, and bridge rehabilitation [100,101,102,103]. However, the introduction of 1% industrial steel fiber doubles the material expense [104]. Thus, the usage of steel fiber derived from used tires has become a viable alternative for the production of SFRC. The performance of concrete reinforced with recycled steel fibers is equivalent to that of industrial SFRC [105,106,107]. This provides an ecologically friendly solution to some of the issues linked with the production of discarded tires [108]. In addition, it assists to promote sustainability in the building sector [109,110,111]. Another challenge to the use of SFRC is corrosion of steel fibers, but coating (brass/zinc/copper) the steel fiber can help resolve this issue [112,113,114]. Furthermore, getting the uniform dispersal of steel fibers in the matrix is another issue related to the use of SFRC. However, using the layer procedure for missing the ingredients of SFRC is the best suitable option to achieve the uniform dispersion of fibers [4,115]. 

## 5. Conclusions

The goal of this study was to carry out a scientometric-based review of the current literature on SFRC research in order to evaluate several criteria. Scopus was searched for 9562 relevant articles, and the records were assessed using the VOSviewer tool. This research yielded the following findings:

- An assessment of publishing sources (journals/conferences), including articles on SFRC research, revealed that “CBM”, Engineering structures”, and “American concrete institute, ACI special publication” are the top three sources, with 877, 420, and 403 publications, respectively. In terms of total citations, the top three publishing sources are “CBM” with 29,863, “Cement and concrete research” with 29,863, and “Cement and concrete composites” with 9708 citations.
- Keywords analysis on the SFRC research revealed that steel fibers, fiber-reinforced concrete, concrete, steel fiber-reinforced concrete, and reinforced concrete are the five most often occurring terms. The keyword analysis found that steel fibers incorporation in concrete has mostly been investigated to improve the mechanical and durability performance of concrete, especially to control the brittleness resisting the crack propagation through cracks bridging and improve ductility.
- Analysis of authors in the current study field showed that 39 authors had published at least 30 articles up to June 2022. Based on the number of publications, total citations, and average citations, the leading researchers were categorized. According to the evaluation, Zhang Y. is the most productive researcher with 86 publications, followed by Yoo D.-Y. with 66 and Li J. with 63 publications. Yoo D.-Y. leads the field in total citations with 2944 overall citations, Banthia N. is second with 2689, and Naaman A.E. is third with 2269 citations in the present research domain. Moreover, when the average number of citations is compared, Naaman A.E. may be at the top with almost 71 average citations, Shah S.P. may be second with about 47, and Yoo D.-Y. may be third with around 45 average citations.
- An evaluation of published articles comprising data on SFRC research discovered that Song P.S.’s [78] paper “Mechanical properties of high-strength steel fiber-reinforced concrete” received 593 citations. Brandt A.M. [79] and Park S.H. [80] received 552 and 406 citations for their articles, respectively, and were among the top three. In addition, as of June 2022, just 60 papers had received more than 200 citations in the topic field.
- The top countries were evaluated on their contribution to SFRC research, and it was revealed that only 35 countries produced at least 50 documents. China, the United States, and India each provided 1607, 1139, and 923 papers. Furthermore, the United States received 24,770 citations, China received 22,924 citations, and Italy received 12,190 citations, and might be placed in the top three based on citations.
- SFRC has been utilized in a variety of engineering applications, including bridges, tunnel linings, retaining walls, road pavements, slabs, hydraulic structures, shotcrete, foundations, and precast members.
- The major challenges to the use of SFRC include high cost, corrosion of steel fibers, and uniform dispersion of fibers in the mix. However, when using recycled steel fibers, especially from the waste tires, coating steel fiber with zinc/brass/copper can be better solutions to these challenges.

## Figures and Tables

**Figure 3:**
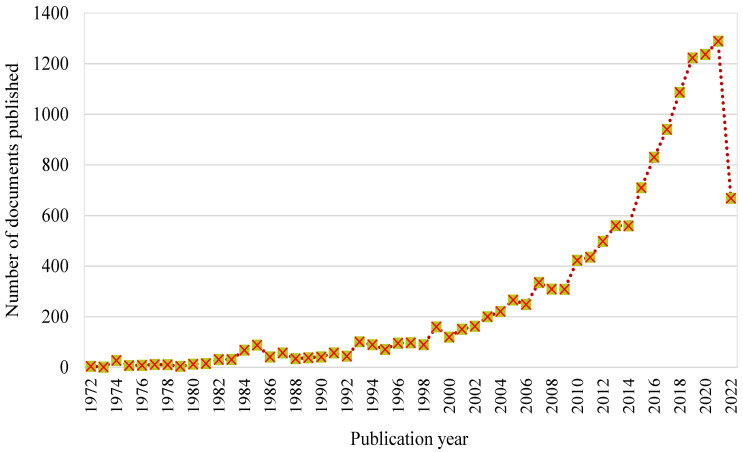
Documents published annually on the SFRC research up to June 2022.

**Table 1:** Sequence of research strategy, selected options, and limits applied at each step.

Step No.		Description	Choice Made	Filters/Limits Used
1		Database selection	Scopus	Subject area: Material science, engineering, and environmental science Source type: Journal and conference proceeding Document type: Journal papers, Journal review, conference paper, and conference review Publication stage: Final Language: English
2		Data mining	CSV files	Abstract and keywords Citation and Bibliographic information Funding details and other information
3		Software selection	VOSviewer	Version 1.6.18
4		Analysis	CSV files to VOSviewer	Type of data: build a map based on bibliographic data Data source: read data from bibliographic database
	4.1	Sources of publications	Bibliographic coupling of sources	Type of analysis: Bibliographic coupling Unit of analysis: Sources Least publication limit for a source: 100
	4.2	Keywords	Co-occurrence of keywords	Type of analysis: Co-occurrence Unit of analysis: Author keywords Least co-occurrence of a keyword: 50
	4.3	Authors	Co-authorship of authors	Type of analysis: Co-authorship Unit of analysis: Authors Least publication limit for an author: 30
	4.4	Documents	Bibliographic coupling of documents	Type of analysis: Bibliographic coupling Unit of analysis: Document Least citations limit for an article: 150
	4.5	Countries	Bibliographic coupling of countries	Type of analysis: Bibliographic coupling Analysis unit: Countries Least publication limit for a country: 50
5		Results and discussions		

**Table 2:** Detail of publication sources with minimum 100 publications up to June 2022.

S/N	Publication Source (Journal/Conference)	Documents Published	Citations Received
1	Construction and building materials	877	29,863
2	Engineering structures	420	8520
3	American concrete institute, ACI special publication	403	1639
4	IOP conference series: materials science and engineering	228	428
5	ACI structural journal	217	7275
6	Materials	209	1569
7	Materials and structures/materiaux et constructions	182	6185
8	Cement and concrete composites	179	9708
9	Composite structures	174	4353
10	ACI materials journal	151	6970
11	Magazine of concrete research	150	2090
12	Journal of materials in civil engineering	148	5462
13	Structures	146	717
14	Structural concrete	142	1484
15	Cement and concrete research	127	9749
16	Journal of building engineering	112	883
17	Materials today: proceedings	105	307

**Table 3:** Detail of 30 mostly utilized keywords in SFRC research.

S/N	Keyword	Occurrences
1	Steel fibers	1393
2	Fiber-reinforced concrete	718
3	Concrete	498
4	Steel fiber-reinforced concrete	477
5	Reinforced concrete	375
6	Ductility	331
7	Compressive strength	315
8	Mechanical properties	308
9	Flexural strength	303
10	Durability	190
11	Shear strength	180
12	Strengthening	175
13	Toughness	168
14	Shear	157
15	Fibers	155
16	Self-compacting concrete	146
17	SFRC	140
18	Strength	138
19	Cracking	135
20	Corrosion	131
21	High-strength concrete	127
22	UHPFRC	111
23	Confinement	108
24	Fiber-reinforced concrete	108
25	steel fiber-reinforced concrete (SFRC)	108
26	High strength concrete	106
27	Tensile strength	105
28	Flexural behavior	94
29	Fiber-reinforced concretes	93
30	Composite materials	92

**Table 4:** Detail of researchers with minimum 30 publications in SFRC studies up to May 2022.

S/N	Author	Number of Publications	Overall Citations	Average Citations
1	Zhang Y.	86	1247	15
2	Yoo D.-Y.	66	2944	45
3	Li J.	63	1481	24
4	Banthia N.	61	2689	44
5	Wang Y.	58	683	12
6	Foster S.J.	52	1240	24
7	Zhang X.	52	629	12
8	Wang Z.	50	454	9
9	Barros J.A.O.	49	1448	30
10	Zhang J.	48	871	18
11	Li X.	48	511	11
12	Liu J.	47	894	19
13	Li C.	46	514	11
14	Plizzari G.A.	44	1139	26
15	Wang J.	44	575	13
16	Gao D.	44	465	11
17	Li Y.	42	430	10
18	Liu Z.	41	636	16
19	Liu X.	41	450	11
20	Wu C.	40	902	23
21	Yoon Y.-S.	38	1525	40
22	Minelli F.	38	851	22
23	Ding Y.	36	923	26
24	Liu Y.	36	272	8
25	Li S.	34	537	16
26	Singh S.P.	33	888	27
27	Nanni A.	33	804	24
28	De La Fuente A.	33	546	17
29	Li Z.	33	461	14
30	Fantilli A.P.	33	459	14
31	Wang X.	33	296	9
32	Naaman A.E.	32	2269	71
33	Hao H.	32	984	31
34	Kim S.	32	642	20
35	Pilakoutas K.	31	726	23
36	Yang Y.	31	212	7
37	Shah S.P.	30	1424	47
38	Meda A.	30	962	32
39	Zhou Y.	30	301	10

**Table 5:** Detail of leading five documents based on citations received up to June 2022.

S/N	Document	Title	Overall Citations
1	Song P.S. [78]	Mechanical properties of high-strength steel fiber-reinforced concrete	593
2	Brandt A.M. [79]	Fiber-reinforced cement-based (FRC) composites after over 40 years of development in building and civil engineering	552
3	Park S.H. [80]	Tensile behavior of ultra-high performance hybrid fiber-reinforced concrete	406
4	Wille K. [81]	Properties of strain hardening ultra-high-performance fiber-reinforced concrete (UHP-FRC) under direct tensile loading	388
5	Yao W. [82]	Mechanical properties of hybrid fiber-reinforced concrete at low fiber volume fraction	382

**Table 6:** Detail of countries published minimum 50 documents up to June 2022.

S/N	Country	Number of Publications	Citations Received
1	China	1607	22,924
2	United States	1139	24,770
3	India	923	8211
4	Canada	539	10,404
5	Australia	471	9633
6	Italy	466	12,190
7	United Kingdom	434	8602
8	South Korea	396	10,384
9	Iran	320	6586
10	Japan	312	4089
11	Spain	293	5216
12	Iraq	266	1533
13	Germany	260	2900
14	Turkey	255	6507
15	Malaysia	180	2328
16	Egypt	171	1151
17	Portugal	166	3902
18	Poland	156	2223
19	Saudi Arabia	152	1928
20	France	143	3558
21	Brazil	135	1733
22	Czech Republic	132	840
23	Singapore	129	3946
24	Hong Kong	113	3026
25	Pakistan	103	1164
26	Belgium	101	1522
27	Switzerland	99	2071
28	Greece	96	2267
29	Russian Federation	89	617
30	United Arab Emirates	88	1186
31	Taiwan	84	2800
32	Netherlands	79	2321
33	Viet Nam	67	1227
34	Sweden	59	1046
35	Argentina	53	1393

## Data Availability

The data used in this research has been properly cited and reported in the main text.
